# Effects of Fluoridated Milk on Root Dentin Remineralization

**DOI:** 10.1371/journal.pone.0104327

**Published:** 2014-08-05

**Authors:** Wolfgang H. Arnold, Bastian A. Heidt, Sebastian Kuntz, Ella A. Naumova

**Affiliations:** Department of Biological and Material Sciences in Dentistry, School of Dentistry, Faculty of Health, University of Witten/Herdecke, Witten, Germany; University of Nebraska-Lincoln, United States of America

## Abstract

**Background:**

The prevalence of root caries is increasing with greater life expectancy and number of retained teeth. Therefore, new preventive strategies should be developed to reduce the prevalence of root caries. The aim of this study was to investigate the effects of fluoridated milk on the remineralization of root dentin and to compare these effects to those of sodium fluoride (NaF) application without milk.

**Methods:**

Thirty extracted human molars were divided into 6 groups, and the root cementum was removed from each tooth. The dentin surface was demineralized and then incubated with one of the following six solutions: Sodium chloride NaCl, artificial saliva, milk, milk+2.5 ppm fluoride, milk+10 ppm fluoride and artificial saliva+10 ppm fluoride. Serial sections were cut through the lesions and investigated with polarized light microscopy and quantitative morphometry, scanning electron microscopy (SEM) and energy-dispersive X-ray spectroscopy (EDS). The data were statistically evaluated using a one-way ANOVA for multiple comparisons.

**Results:**

The depth of the lesion decreased with increasing fluoride concentration and was the smallest after incubation with artificial saliva+10 ppm fluoride. SEM analysis revealed a clearly demarcated superficial remineralized zone after incubation with milk+2.5 ppm fluoride, milk+10 ppm fluoride and artificial saliva+10 ppm fluoride. Ca content in this zone increased with increasing fluoride content and was highest after artificial saliva+10 ppm fluoride incubation. In the artificial saliva+10 ppm fluoride group, an additional crystalline layer was present on top of the lesion that contained elevated levels of F and Ca.

**Conclusion:**

Incubation of root dentin with fluoridated milk showed a clear effect on root dentin remineralization, and incubation with NaF dissolved in artificial saliva demonstrated a stronger effect.

## Introduction

The positive influence of fluoride application in enamel caries prevention is now widely accepted. The preventive effect of fluoride on caries is dependent on the dose and the local effect of fluoride on the tooth surface [1]. A number of studies have demonstrated the effectiveness of topical fluoride application, such as dentifrices, fluoride gels, varnishes and mouth rinses, in the prevention of coronal caries [2], [3]. The value of alternative fluoride applications, such as water and milk fluoridation, is still being discussed, although a number of studies are available on the preventive effects of these alternative methods of fluoride application [4]. Very few clinical studies exist on the effectiveness of fluoridated milk in caries prevention [5]–[7]. Yeung et al. [8] identified only 2 randomized clinical trials that reported a reduction of the DMFT (Decayed, Missing, Filled Teeth) in populations that received fluoridated milk compared with populations that did not. However, the results of those two studies were controversial [6], [7]. Studies on the preventive effect of fluoride have in common that they address coronal caries.

With the changing demographics in industrialized countries and the advent of effective preventive dental health programs, more elderly people retain their teeth but often suffer from periodontitis and gingival recession. Both factors are correlated with an increasing prevalence of root caries [9]–[11]. Therefore, the occurrence of root caries is becoming an important health problem among elderly people, and new preventive strategies are necessary to reduce this problem. To date, only a few papers have addressed the question of how to reduce the prevalence of root caries [12], [13], but milk fluoridation has been shown to be effective in the prevention of enamel caries [4], [6]–[8], [14]–[16]. Milk is easily accessible and seems to be an ideal vehicle for fluoride administration. Furthermore, milk is rich in Ca, which may also enhance the remineralization process. The preventive effects of fluoride are dependent on its bioavailability, and the effects vary considerably after administration due to variations in fluoride formulation and rates of salivary flow [3], [17], [18]. Shortly after fluoride administration with dentifrice, the fluoride concentration in the saliva increases rapidly but returns to the baseline level after 120 minutes [18]. Therefore, to receive the most benefit, fluoride may need to be administered in small portions throughout the day.

Because of fundamental structural differences between enamel and dentin, fluoridated milk may not have the same positive effects on dentin remineralization as it does on enamel. The aim of this study was to investigate the effects of fluoridated milk on root dentin remineralization and to compare these effects with those of the application of NaF in artificial saliva. The null hypothesis of this study was that there would be no difference between the effects of fluoridated milk and NaF in saliva on root dentin remineralization.

## Materials and Methods

### Experimental procedure

For the present investigation, 30 premolars that had been extracted for orthodontic reasons and were free of root caries were divided into 6 groups of 5 teeth each. Patients provided their verbal consent to use the teeth for this *in vitro* study. The protocol conformed to the principles outlined in the Central German Ethics Committee's statement (2003) focusing on the use of human body material in medical research and was approved by the Ethical Committee of Witten/Herdecke University (116/2013). According to the ethics committee statement, written consent was not necessary as the teeth were used anonymously. In the case of minors, consent was obtained from the parents or caretakers with the agreement of the children. Verbal consent was documented in the patient file. The exclusion criterion for use of the teeth was the presence of periodontal diseases. The teeth were stored in 0.9% NaCl containing 0.1% thymol. The root cementum within the cervical area of the buccal or vestibular surfaces was removed using a diamond burr under a stereomicroscope to ensure that the cementum was removed completely, and the roots were covered with dental wax so that a 4×4 mm window remained on the root surface. These windows were then demineralized in 1.6% hydroxyethylcellulose acidified with acetic acid at pH 4.7 for 3 days [19].

After demineralization, the teeth were incubated at 37°C for 7 days in 20 ml of various remineralizing solutions in closed plastic bottles at pHs between 6.8 and 7.0. The incubation media were freshly prepared and changed every 24 hours. The pH was checked before and after 24 hours incubation. Before incubation in the new medium, the teeth were rinsed with distilled water for 1 minute. The different incubation media and their contents are summarized in Table 1.

**Table 1:** Composition of incubation media.

Incubation medium	Content	
NaCl	0.9%	
Milk	whole milk 3.5% fat	
Artificial saliva	KCl	150 mmol/L
	CaCl_2_	1.5 mmol/L
	KH_2_PO_4_	0.9 mmol/L
	Sodium acetate buffer	0.1 mol/L
Milk+2.5 ppm	whole milk 3.5% fat	
	2.5 ppm fluoride from NaF	
Milk+10 ppm	dto.+10 ppm fluoride from NaF	
Artificial saliva+10 ppm	same as in # 3+10 ppm fluoride from NaF	

After the dental wax was removed, the roots were embedded into Technovit 9100 (Kulzer, Wehrheim, Germany), and serial sections with a thickness of 80 µm were cut through the experimental lesions. Three sections from the center of each lesion were selected for further evaluation.

### Polarized light microscopy

Light microscopic analyses of the sections were conducted using polarized light microscopy (PLM) (Leica DMRB, Leitz, Wetzlar, Germany) and quantitative morphometric measurements. All sections underwent visual evaluation and qualitative analysis. The depth of the demineralized zone was measured quantitatively from the surface to the bottom of the lesion, and the data were compared statistically. Ten single measurements of the depth of the demineralized zone were determined in each section.

### Scanning electron microscopy (SEM) and energy-dispersive X-ray spectroscopy (EDS) analysis

**Figure 1:**
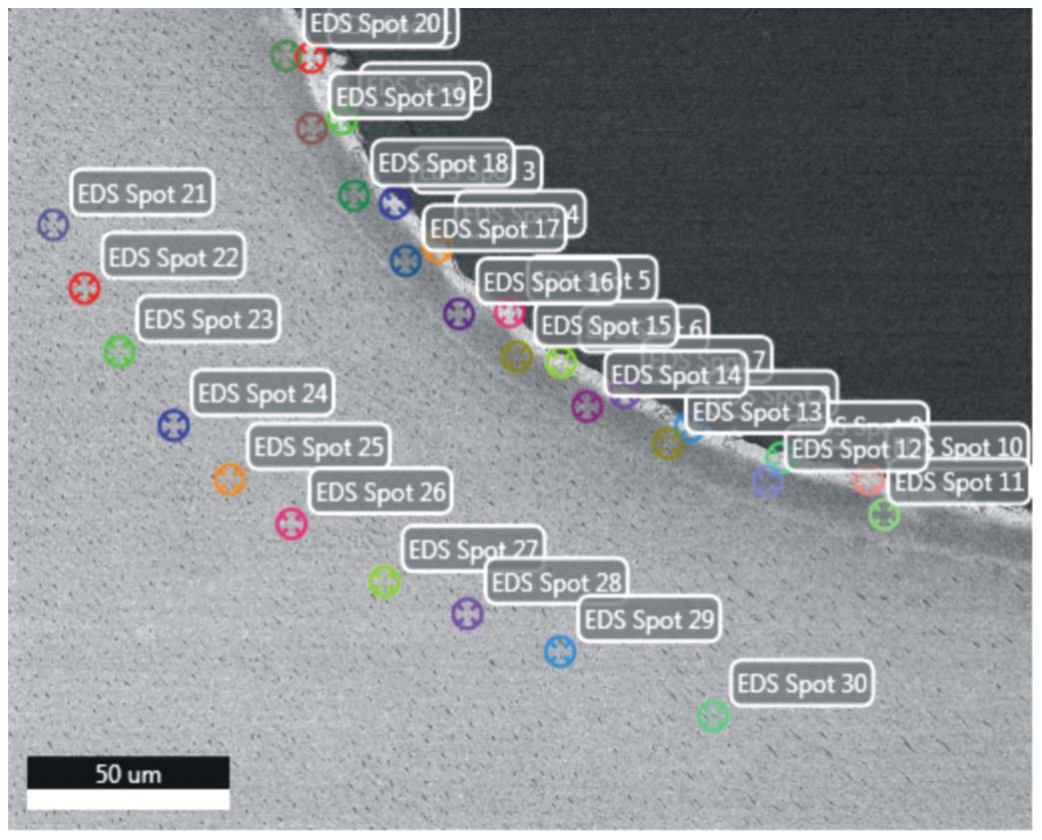
Distribution of the locations of the spot measurements. Three rows (surface, demineralized zone and sound dentin) of 10 points each were selected for quantitative element analysis.

The same sections underwent an evaluation with SEM combined with quantitative EDS. SEM was conducted using a Zeiss Sigma VP SEM at a pressure of 20 Pascal and with 20 kV accelerating voltage and an AsB backscattered electron detector. EDS was conducted with an EDAX Apollo XL (EDAX Inc., Mahwah, NJ, USA) system with an active area of 30 mm^2^ and Team V3.3 software. Three different measurements were recorded: quantitative point measurements, line scans and EDS elemental mapping. For the quantitative point measurements in each lesion, 5 areas were randomly selected. In each area, 3 different zones were determined, including the surface zone, the middle of the lesion and the sound dentin. In each zone, 10 single point measurements were quantitatively analyzed for Ca, P, C and F content (Fig. 1). A quantitative point measurement was conducted at a count rate of 6000–8000 counts per second and with dead time of 10–15% for 30 live seconds. The line scans were acquired with a dwell time of 25 msec and an amplification time of 12.8 µsec, with a distance between each reading point of 1 µm. EDS mappings were acquired with a dwell time of 200 µsec per reading point and a frame resolution of 512×400 pix. A total of 512 frames were recorded, which resulted in a reading time of approximately 8 hrs.

### Statistics

Prior to the experiments, a power analysis was calculated using data from test experiments with two teeth incubated with NaCl and two teeth with fluoridated milk+100 ppm fluoride, on the basis of mean values and standard deviations, (NaCl; mean = 128.66±16.28: milk+100 ppm fluoride; mean = 64.57±10.22; delta = −64.08) and a power of 0.8 with α = 0.05. The power analysis revealed a minimum sample size of 3 teeth. Therefore, 5 teeth per group were used. The mean values per section were calculated from the measurements of the depths of the lesions. The normality of the distributions was tested with the Kolmogorov-Smirnov Test. The means were then compared statistically using one-way ANOVA for multiple comparisons. Quantitative analysis of the point measurements of EDS analysis was calculated using multivariate analyses for repeated measurements. As no intrinsic differences were found, a one-way ANOVA for multiple comparisons was performed for these data. For all statistical tests, post hoc Bonferroni adjustments of the p values were applied. Confidence intervals of 95% were used, and p values of less than 0.05 were considered to be statistically significant.

## Results

### Polarized light microscopy

**Figure 2:**
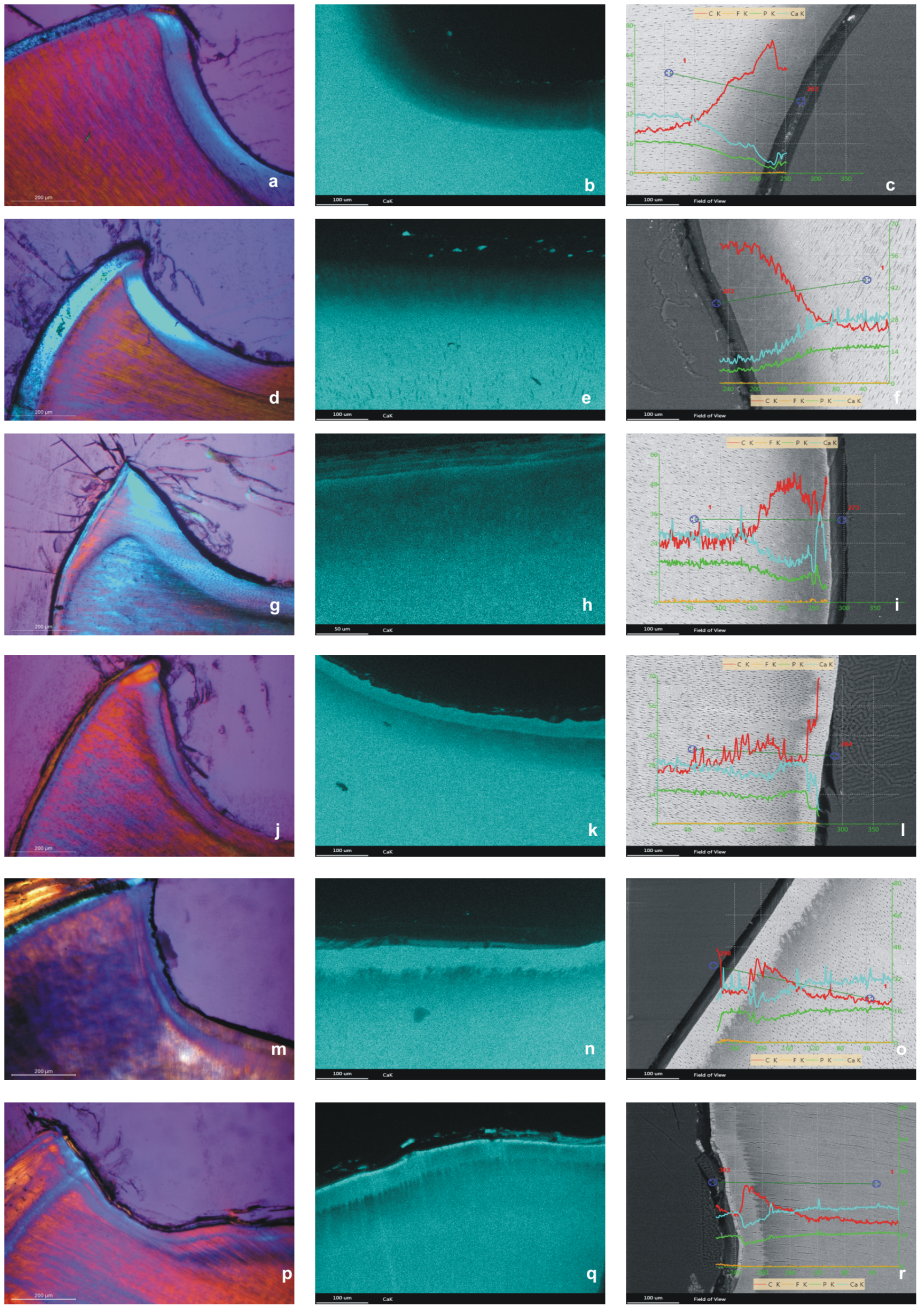
Experimental root caries lesions after different incubation experiments. An experimental root caries lesion after incubation with NaCl solution (a–c). a) A PLM micrograph reveals a homogenous lesion. b) The EDS mapping of Ca reveals a continuous demineralization toward the lesion surface. c) A line scan reveals the continuous demineralization. An experimental root caries lesion after incubation with whole milk (d–f). d) A PLM micrograph reveals a homogenous lesion. e) The EDS mapping of Ca reveals a continuous demineralization toward the lesion surface. f) A line scan reveals the continuous demineralization. An experimental root caries lesion after incubation with artificial saliva (g–i). g) A PLM micrograph reveals a homogenous lesion. h) The EDS mapping of Ca reveals a small remineralized zone on top of the lesion. i) A line scan of the lesion confirms the small remineralized zone. An experimental root caries lesion after incubation with milk containing 2.5 ppm fluoride (j–l). j) The PLM micrograph reveals a carious lesion divided into two layers: the demineralized zone and a clearly demarcated superficial remineralized zone. k) The EDS mapping of Ca reveals increased Ca content in the superficial remineralized zone. l) A line scan demonstrates the superficial remineralized zone above the demineralized lesion. An experimental root caries lesion after incubation with milk containing 10 ppm fluoride (m–o). m) The PLM micrograph reveals a carious lesion divided into two layers: the demineralized zone and a relatively thick superficial remineralized zone. n) The EDS mapping of Ca reveals a remineralized zone with increased Ca content. o) A line scan of the lesion confirms the increased Ca content in the superficial remineralized zone. An experimental root caries lesion after incubation with artificial saliva containing 10 ppm fluoride (p–r). p) The PLM micrograph demonstrates three distinct lesion zones: the demineralized zone, the superficial remineralized zone and an amorphous layer on top of the lesion. q) The EDS mapping of the Ca^2+^ content reveals that the demineralized lesion is divided into two zones by a remineralized zone. On top of the lesion is an amorphous, highly mineralized layer. r) A line scan confirms the scanning results by revealing a remineralized zone in the middle of the demineralized lesion and a highly mineralized zone with increased F^−^ content on top of the lesion.

**Figure 3:**
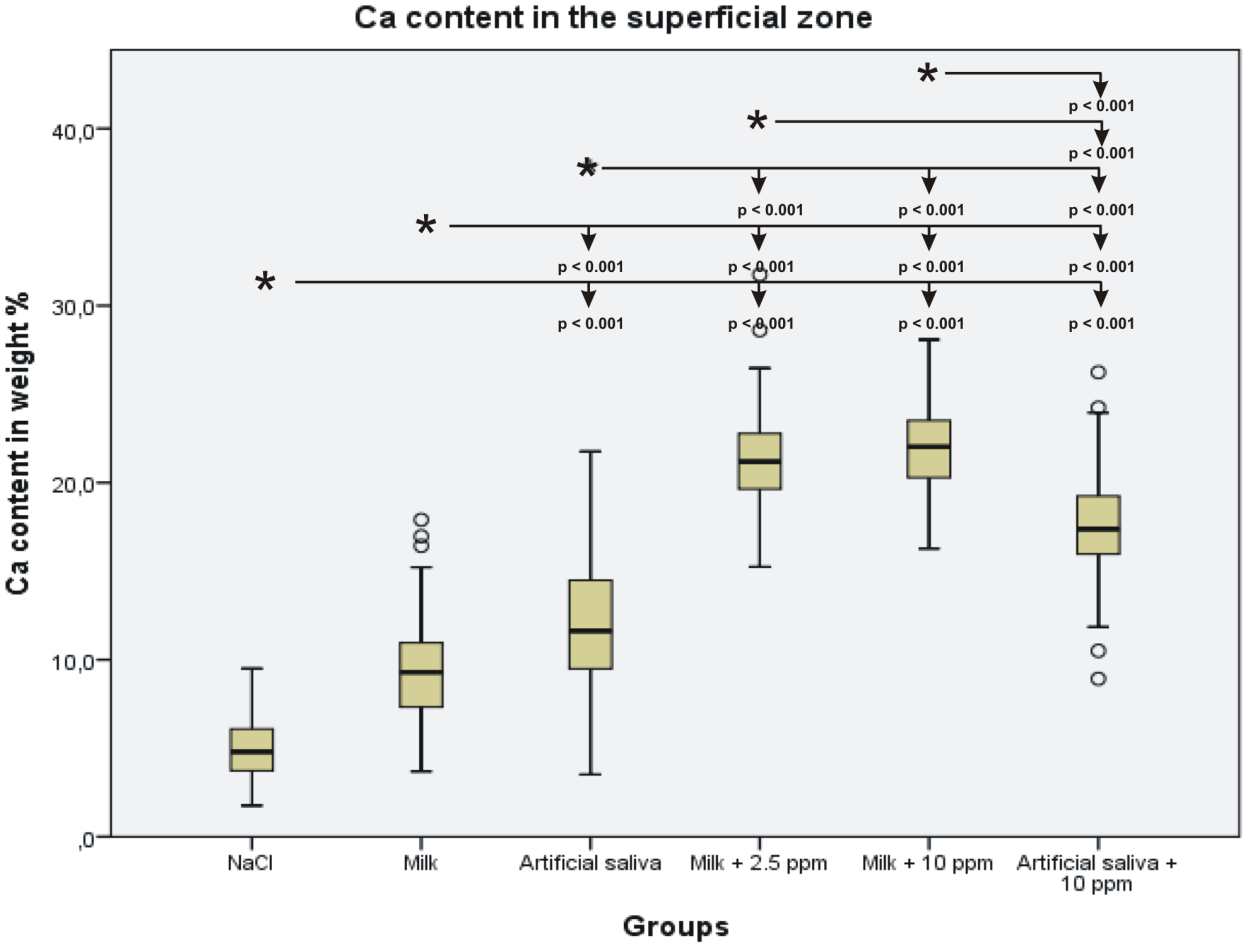
Boxplot graph of the depths of the lesions. The depth of the lesion decreases with increased concentration of F in the incubation medium. The depth is the smallest after incubation with artificial saliva containing 10(p<0.05) between the incubation media are marked with an asterisk, and other media are indicated with arrows.

A clearly marked demineralized zone was found in all experimental lesions. Depending on the type of remineralization solution applied, the depth of the demineralized zone varied. The NaCl control specimens exhibited a large homogeneous demineralized zone (Fig. 2a) with a mean depth of 96.1±13.3 µm. After incubation with milk, there was a homogeneous demineralized zone (Fig. 2d) with a mean depth of 93.5±20.7 µm. When incubated with artificial saliva alone, the specimens showed a homogenous demineralized zone (Fig. 2g) with a mean depth of 116.8±22.6 µm. Incubation with milk containing 2.5 ppm fluoride resulted in a demineralized zone with a mean depth of 91.9±12.6 µm. Notably, this demineralized zone in was divided into two layers of birefringence (Fig. 2j). In the group that was incubated with milk containing 10 ppm fluoride, a mean depth of 73.5±11.9 µm was measured. The demineralized zone in these specimens was also divided into two layers of birefringence (Fig. 2m). After incubation with artificial saliva containing 10 ppm fluoride, three different zones were found: a smaller demineralized zone, a superficial remineralized zone and a crystalline amorphous layer on top of the superficial remineralized zone (Fig. 2p) The mean depth of the carious lesion was 29.3±3.6. The statistical results are summarized in Fig. 3.

### SEM and EDS analysis

SEM revealed clearly demarcated zones of sound dentin, demineralized dentin and remineralized dentin. In the NaCl group, the EDS elemental mapping of Ca, P and C showed a continuous decrease in Ca and P content from sound dentin toward the surface area and an increase in C content (Fig. 2b). This was confirmed by the line scan through the lesion (Fig. 2c). In the milk group, similar mapping results were found, with a continuous decrease in Ca and P content and an increase in C content (Fig. 2e). The line scan confirmed these results (Fig. 2f). EDS mapping of the artificial saliva group revealed a small remineralized surface zone (Fig. 2h) that was verified in the line scan (Fig. 2i). After incubation with milk containing 2.5 ppm fluoride, a superficial remineralized zone with a mean thickness of 29.3±3.6 µm was found. In this zone, elevated Ca and P contents were observed in both the elemental maps and the line scans (Fig. 2k and l). A similar remineralization pattern was found after incubation with milk containing 10 ppm fluoride (Fig. 2n and o). However, the superficial remineralized zone was larger than that in the milk+2.5 ppm fluoride group, as the mean thickness was 31.9±5.1 µm. The difference was statistically significant (p = 0.001). A different pattern of elemental distribution was found after incubation with artificial saliva containing 10 ppm fluoride. A highly mineralized layer was found on top of the lesion, showing a crystalline structure that did not resemble normal dentin. Underneath this crystalline layer, a demineralized zone was identified over a superficial remineralized zone (Fig. 2q). The line scan indicated an increased Ca content in the superficial remineralized zone of the lesion and relatively high Ca content in the surface layer on top of the lesion. This layer also contained elevated levels of fluoride content, which was confirmed by the EDS analysis (Fig. 2r). The mean Ca and P values were between 4.03 and 24.67 wt% for Ca and 2.2 and 10.92 wt% for P. All quantitative results of the superficial remineralized zone are summarized in Table 2.

**Table 2:** Results of the quantitative point measurements in the superficial remineralized zone in weight %.

Incubation medium	Ca^2+^ in wt%		P^5−^ in wt%		F^−^ in wt%		Ca/P ratio	Ca/F ratio
	mean	standard dev.	mean	standard dev.	mean	standard dev.		
NaCl	4.03	0.52	2.20	0.23	0.20	0.10	1.83	19.86
Milk	10.35	1.28	4.85	0.56	0.29	0.16	2.13	36.16
Artificial saliva	14.84	2.26	7.86	1.60	0.57	0.21	1.89	25.96
Milk+2.5 ppm F	20.87	2.07	9.81	0.84	0.83	0.20	2.13	25.29
Milk+10 ppm F	21.87	1.01	10.24	0.35	1.58	1.15	2.14	13.84
Artificial saliva+10 ppm F	23.61	1.50	10.90	0.60	2.08	0.36	2.17	11.35

**Figure 4:**
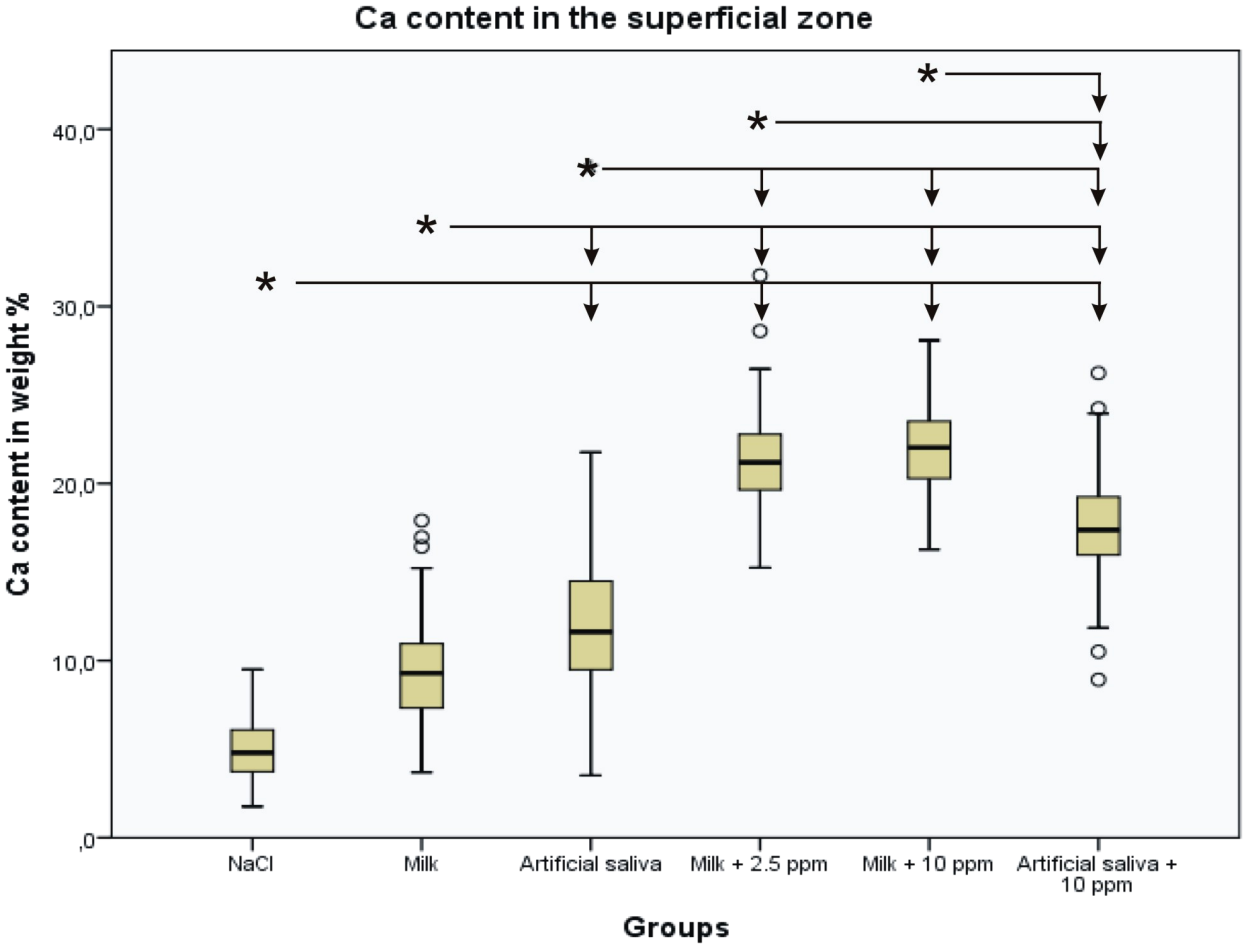
Boxplot graph of the Ca content in the superficial remineralized zone of the lesion. The Ca^2+^ content increases with increased Ca concentration in the incubation medium. Significant differences (p<0.05) between the incubation media are marked with an asterisk, and other media are indicated with arrows.

**Figure 5:**
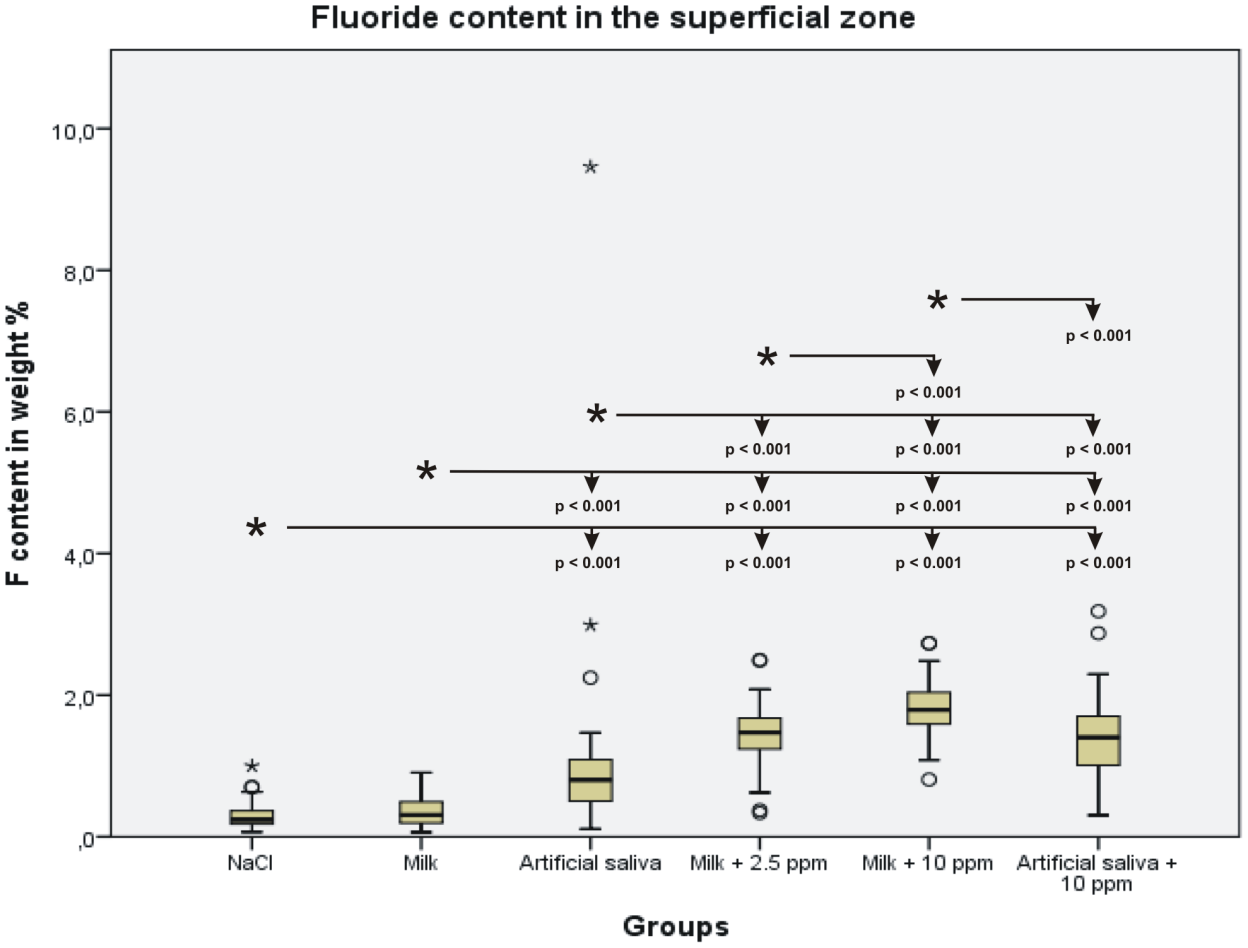
Boxplot graph of the F content in the superficial remineralized zone of the lesion. The F content increases with increased F concentration in the incubation medium. Significant differences (p<0.05) between the incubation media are marked with an asterisk, and other media are indicated with arrows.

Statistical analysis of the Ca and F content showed an increase of Ca and F content in the superficial remineralized zones with increasing F^−^ concentration in the incubation medium The differences in Ca content in the superficial zone were statistically significant between the NaCl control group and all other groups, but the difference between the NaCl group and the milk group was not statistically significant. The statistical results of the Ca measurements are summarized in Fig. 4. The F content increased accordingly. Significant differences were found between the NaCl control group and all other groups, except for the milk group (Fig. 5). The Ca and F content in the crystalline superficial layer on top of the lesion resembled the content of those elements in the superficial remineralized zones of the other groups. However, the Ca/F ratio in this layer was 42.2, which indicates lower F content relative to Ca content compared with the superficial remineralized dentin zone of the same group.

## Discussion

For decades, PLM has been used for the investigation of enamel and dentin caries lesions [19]–[24]. It allows for determination of the morphological structure of lesions, as well as differentiation between differently mineralized zones. In combination with quantitative EDS elemental analysis, PLM has been demonstrated to be a good tool for the investigation of demineralization and remineralization [16], [19], [20], [25], [26].

Enamel caries lesions and root caries lesions are quite different in their morphology and pathogenesis [10]. The etiologic mechanisms of enamel caries are well understood [27], but the etiology of root caries lesions remains unclear [11], [28], [29]. The dental root is covered by a thin layer of cementum that can be penetrated by bacteria or dissolved by acids, resulting in open dentin tubules and hypersensitive teeth [9], [30].

Initially, coronal caries lesions have a well-mineralized surface layer [21]. This layer is absent in root caries lesions, in which dentin, which is predominantly demineralized, is typically present on the surface [10], [19], [20]. In some natural root caries lesions where the cementum is still present, a small hypermineralized superficial cementum layer can be found [10]. In this respect, natural root caries lesions can be different from artificial carious lesions. On the other hand, natural root caries lesions are highly individual and cannot be standardized. In this study, the root cementum was mechanically removed before the experiments. Therefore, root dentin was always at the surface zone of the experimental lesions. Standardized demineralization resulted in standardized and comparable root carious lesions.

A number of studies have investigated the effectiveness of fluoridated milk on enamel caries, and fluoridated milk has been shown to have a positive effect on caries reduction and enamel remineralization [4], [5], [15], [25], [31]–[34]. Only a few studies have assessed the effect of fluoridated milk on dentin remineralization [13], [35]. The present study also showed that fluoridated milk has an effect on dentin remineralization. This effect is clearly dose dependent, which is consistent with previous findings of a dose-dependent effect on enamel [8], [12], [13], [35]. However, one study did not support this assumption [15], instead showing that milk with 2.5 ppm or higher concentrations of fluoride had the same effect. A direct influence of the other components of milk, such as other minerals or proteins, is unlikely because incubation with milk alone showed no remineralizing effects. However, the influence of proteins, fat and other minerals in milk on the bioavailability of fluoride cannot be excluded, but was not considered in this study. A limitation of this study is that it did not mimic the daily clinical situation and the probable influences of biofilm on demineralization and remineralization of the root surface. This question deserves further study. The newly built mineral on the root dentin surface of experimental lesions appears to be impure hydroxyapatite, as suggested by the Ca/P ratio. In the lesions that were incubated with NaCl or artificial saliva, the Ca/P ratio was within the range of hydroxyapatite, whereas after incubation with fluoridated milk or fluoride-containing artificial saliva, the Ca/P ratio was outside of this range. This indicated that the remineralization product of dentin is a mixture of different calcium phosphate minerals. Further investigations are needed to elucidate the solubility of the remineralized dentin zone and the function of this zone in preventing further root caries progression.

In this study, artificial saliva containing 10 ppm F was used to compare with the remineralizing effect of fluoridated milk. This F concentration represents the salivary fluoride concentration thirty minutes after tooth brushing with F-containing toothpaste. Two hours after tooth brushing, the saliva fluoride level has usually returned to baseline [18]. Milk is usually applied irregularly and remaining a relatively short time within the oral cavity before being swallowed. Therefore, the fluoride from fluoridated milk may have a rather limited effect on fluoride bioavailability.

The SEM results also demonstrated that the remineralization effect of fluoridated milk is restricted to the demineralized dentin surface. Fluoridated milk may have a positive effect on the remineralization of demineralized dentin surfaces. In contrast, after remineralization with fluoridated artificial saliva, a newly formed crystallite layer covered the surface. The Ca/P ratio indicated that this layer was not pure hydroxyapatite; rather, based on the elevated F^−^ content, the layer was a mixture of different calcium phosphate minerals, including fluorapatite.

Limitations of the present study are the *in vitro* design of the experimental approach and the permanent incubation of the teeth in the remineralization medium. The incubation approach does not reflect reality, as remineralizing agents in the oral cavity only temporarily cover the root surface when they are administered and shortly thereafter. This may explain the higher Ca and F content in the remineralized zone of the specimens that were incubated with fluoridated milk compared with the specimens incubated with artificial saliva+10 ppm fluoride.

Based on the results of the present study, fluoridated milk may be another helpful tool in root caries prevention. However, the effects of fluoridated milk are limited. As this study was an experimental *in vitro* study, the clinical effects of fluoridated milk remain to be investigated.

## Conclusions

Fluoridated milk appears to have a positive, dose-dependent effect on root dentin remineralization. However, the effect of artificial saliva was even stronger. Therefore, the null hypothesis of the present study was rejected.
