# Correction: Effects of Fluoridated Milk on Root Dentin Remineralization

**DOI:** 10.1371/journal.pone.0108199

**Published:** 2014-09-09

**Authors:** The PLOS ONE Staff

Figure 3 is an incorrect duplicate of Figure 4. The authors have provided a correct Figure 3 here.

**Figure 3:**
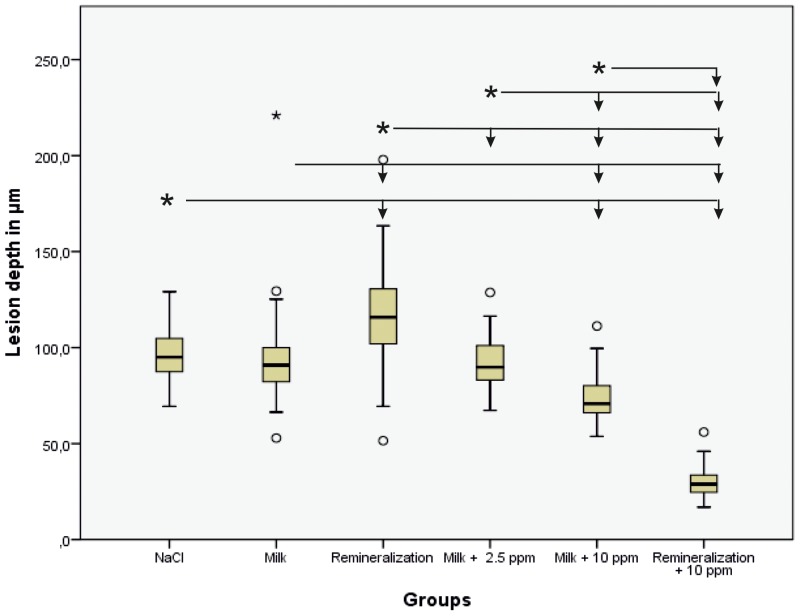
Boxplot graph of the depths of the lesions. The depth of the lesion decreases with increased concentration of F in the incubation medium. The depth is the smallest after incubation with artificial saliva containing 10(p<0.05) between the incubation media are marked with an asterisk, and other media are indicated with arrows.
